# A Self-Adapting, Pixelized Planar Antenna Design for Infrared Frequencies

**DOI:** 10.3390/s22103680

**Published:** 2022-05-12

**Authors:** Mustafa Shubbar, Balázs Rakos

**Affiliations:** Department of Automation and Applied Informatics, Budapest University of Technology and Economics, 1117 Budapest, Hungary; shubbar@aut.bme.hu

**Keywords:** infrared antenna, pixel antenna, self-adapting antenna, bolometer, infrared sensor, infrared energy harvesting

## Abstract

Infrared antennas with reconfigurable characteristics offer several advantages in the medical, military, telecommunication and energy harvesting areas, while their design and implementation is a particularly challenging task for the researchers. This paper proposes a pixel antenna, designed for mid-infrared frequencies with a bandwidth more than 25 THz, consisting of 3 × 3 square metallic planar patches. Bolometer-based switches are placed between the adjacent pixels in order to obtain the adaptable characteristics, optimized for the incoming infrared radiation. The incident wave from a certain direction will heat up the bolometers. Consequently, the conductivity of these bolometers (PTC) will be decreased, and as a result they can be considered to turn to OFF state. The simulation results suggest that the proposed structure can steer the antenna pattern toward the direction of the incident radiation in an adaptable manner, thereby considerably increasing the antenna gain. The gain of the antenna can be increased up to 2 dB with respect to the reference one, which makes it a promising structure for various applications.

## 1. Introduction

### 1.1. Application of Antennas in Infrared Detectors

The demand for using infrared (IR) detectors has increased dramatically in the last few decades with respect to several applications including military, space science, medicine and energy harvesting [1]. Generally, the sensors can be classified into two groups: Photon detectors take advantage of the photonic nature of the incident light by usually utilizing quantum-mechanical effects arising in semiconductors, and on the other hand, thermal detectors can be either bolometer-based or thermoelectric. In the case of thermal sensors, the absorbed radiation changes the temperature of their material, thereby its resistance. The output signal of such devices doesn’t depend on the photonic nature of the incident radiation [2,3].

The development of IR detectors, especially in the case of longer wavelength radiation (e.g., mid-IR range), faces several challenges related to the cooling requirements, spectral response, cut-off frequency issues, responsivity and size. The incorporation of IR antennas in such sensors offers a potential solution for such issues, while offering excellent control on the frequency and spatial domains, which is not directly available in the case of conventional IR sensors. Additionally, the antenna-coupled structures combine the benefits of the spatially compact sensor and large collection area, which is independent of the sensor area. Furthermore, such integrated devices can be operated at room temperature [3]. Bonakdar et al. [4] (pp. 142–145) demonstrated that optical antennas can improve light–matter interaction in quantum structures, like quantum wells, and boost the responsivity of these detectors. J. Gou et al. [5] (p. 91) suggested that spiral antennas can significantly improve the tunable THz-frequency absorption of microbridge structures and provides an effective way to fabricate THz-frequency microbolometer detectors with great potential in real-time imaging applications. L. A. Florence et al. [6] (pp. 1299–1301) used a V-shaped, linear, tapered slot antenna (V-LTSA), coupled to a metal-oxide-metal (MOM) diode, characterized at 10.6μm wavelength. Since it offers promising characteristics for IR radiation sensing, it is expected to become a desirable sensor for broadband IR detection. B. A. Slovick et al. [7] (pp. 119–122) demonstrate a MOM diode as detector element with a considerable improvement in angular resolution with the aid of a four-element phased array antenna versus that of two array elements. They found that the effective antenna aperture can be made larger with a narrower angular resolution by increasing the number of antenna elements with fixed separation distances. K. Kim et al. [8] (pp. 196–205) reported an improved detectivity seven times more than that of conventional microbolometers by coupling the sensors with 3D feed horn antennas. J. Horikawa et al. [9] (pp. 21–24) observed the improvement and outstanding polarization dependence of superconducting mid-IR detectors as a result of the antenna properties. T. Morf et al. [10] (pp. 96–104) presented a new sensor concept based on an antenna-coupled MOSFET bolometer, suitable for room temperature operation. A broad-bandwidth THz antenna absorbing the electromagnetic field is directly coupled to a bolometer for maximizing the energy collection. This design also minimizes the thermal mass, which is essential for a fast frame rate in the case of security and medical applications.

Our proposed antenna arrangement adds further improvements by improving both the gain and bandwidth due to its self-adapting nature that optimizes the antenna to the properties of the incoming radiation. This will be demonstrated in the following sections through simulations.

### 1.2. Antenna-Based Infrared Energy Harvesting

Solar radiation is considered a primary source of energy since it reaches the surface of the earth with a power density of 1370 $W/m^{2}$. The solar spectrum consists of about 52% IR, and 39% visible light energy; the rest of the spectrum contains 9% ultraviolet (UV) radiation [11,12]. The utilization of this type of renewable energy has opened a new era for the production of clean electricity [11]. Since the first application of photovoltaics-based technology in 1950, its utilization is continuously increasing.

Certain studies state that the concentrated IR and UV radiation might decrease the efficiency and shorten the lifetime of solar cells due to the elevated temperature of the cells [13]. Therefore, Khoshdel et al. proposed a plasmonic, cross-shape nanoantenna as IR and UV filter in solar cells. The proposed nanoantenna can block 79.6% and 65.2% of the incident UV and IR radiation, respectively, improve the power efficiency of both silicon and organic solar cells in long term applications and increase their lifetime [14].

Photovoltaics (PV) provides a convenient method for the direct conversion of sunlight into electricity. However, the main drawbacks of conventional, semiconductor-based PV cells are their low energy conversion efficiency, the high cost of materials and the difficulty of the fabrication process. Furthermore, they can’t utilize the mid-IR portion of the solar spectrum, are sensitive to the weather conditions and their efficiency deteriorates rapidly with increasing temperature. The application of mechanical sun-tracking systems is essential to achieve maximum conversion efficiency [11,14,15]. Therefore, recently, the application of nanoantennae has gained much interest regarding solar and IR energy harvesting systems, as a potential substitute to traditional solar energy harvesting processes [9,14]. Antenna-based structures are advantageous for such purposes, since they are widely available in various forms and can be manufactured using inexpensive and simple fabrication processes compared to conventional solar cells. Furthermore, they can achieve significantly higher efficiency than solar cells, and can utilize the environmental IR radiation available 24 hours a day, such as heat [10,14].

**Figure 1:**
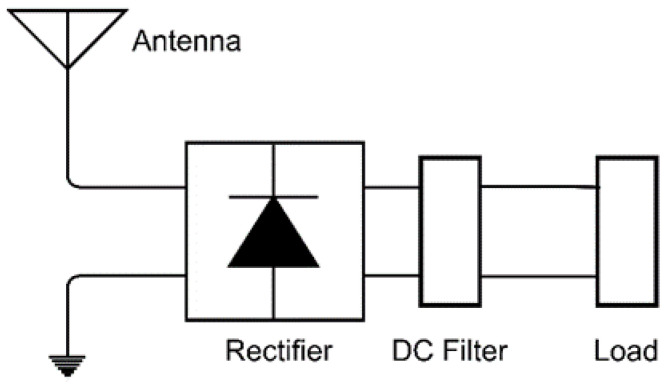
Block diagram of the energy harvesting system based on the rectenna principle.

An infrared rectenna system can harvest electromagnetic radiation by rectifying the currents induced in the antenna with the aid of a high-speed rectifier [10,16,17]. The block diagram of the arrangement is displayed in Figure 1. Its overall efficiency depends on the efficiencies of the individual parts, and the matching level between them [16]. Therefore, many researchers focused their efforts on the antenna gain, matching the level and rectification process. R. Citroni et al. [17] (p. 152918) demonstrated a dynamic impedance matching between an optical rectenna array and the load in order to mitigate the crucial mismatching problem and enhance the conversion efficiency, hence maximizing the captured power.

G. Jayaswal et al. [18] (pp. 1–9) and A. M. A. Sabaawi et al. [11] (pp. 1975–1978) introduced a bowtie antenna for IR field capture, and they proposed a metal–insulator–metal (MIM) diode for rectification of high-frequency currents. Other studies suggest a different rectification approach to overcome the matching problems. G. P. Szakmany et al. [19] (pp. 44–49) and F. J. González et al. [20] (pp. 1837–1842) proposed antennas coupled to thermoelectric junctions. The antenna currents produced by the received IR radiation heats up the hot junction of the thermocouple, thereby creating an electric potential by the Seebeck effect. Nanoscale junctions have a small thermal mass and can be operated much faster than bolometers. On the other hand, A. Chekin et al. [21] (pp. 412–416) proposed nanoantennae placed in an array to achieve a high electric field. The same study suggests a different rectification process related to electron field emission from sharp edges in vacuum, based on the Fowler–Northeim (FN) theory.

Since wide bandwidth, high gain and directivity for THz sensing is required in various applications, and maximum power handling is crucial in energy harvesting systems, an unbiased, reconfigurable antenna with adapting features is a proper solution for the maximization of the power handling, multiple resonance frequencies, high gain and low interference. S. Poorgholam-Khanjari et al. [22] (p. 126482) demonstrate Vivaldi antenna for THz sensing application to obtain the reconfigurable characteristics. They propose the usage of graphene slabs to improve the antenna bandwidth. To overcome the reduced gain and return loss caused by graphene slabs, they propose the application of a hyperbolic metamaterial lens to concentrate the electrical field with an improved gain. F. B. Zarrabi et al. [23] (pp. 34–39) added a graphene coating to the cross-shaped nano-aperture to achieve reconfigurable behavior. Moreover, M. Bazgir et al. [24] (pp. 127–133) improved the characteristics of nanoantennae by the development and application of a unique metamaterial, and their reconfiguration is achieved by using a graphene layer coating.

Although various studies proposed antenna reconfiguration by adding a graphene layer, this method reduces the gain and bandwidth and adds to the manufacturing complexity and cost [22]. However, our proposed structure can achieve the reconfigurable characteristics with improved bandwidth, gain and radiation characteristics.

## 2. Reconfigurable Pixel Antenna Concept

Traditional wireless systems usually have a specific, predefined purpose. Therefore, the antennas in such systems possess fixed parameters regarding both the frequency and spatial domains [25]. Recently, reconfigurable antennas have gained considerable research interest in multi-standard application, and in cases where there is a need for continuous optimization of the antenna performance [25,26]. For example, by manipulating the antenna characteristics one can suppress excessive noise and avoid jamming signals, hence maximizing the power transfer [27].

The reconfiguration process can be done by altering the physical or electrical parameters of the antenna with the aid of internal mechanisms in order to redistribute the RF currents in it [28]. Frequency reconfigurable antennas can change their operating frequency, while radiation-reconfigurable antennas are used for manipulating the radiation characteristics [29,30]. On the other hand, the simultaneous alteration of the frequency and radiation properties is known as compound reconfiguration. Such antennas are suitable for smart communication systems [27,31,32,33].

**Figure 2:**
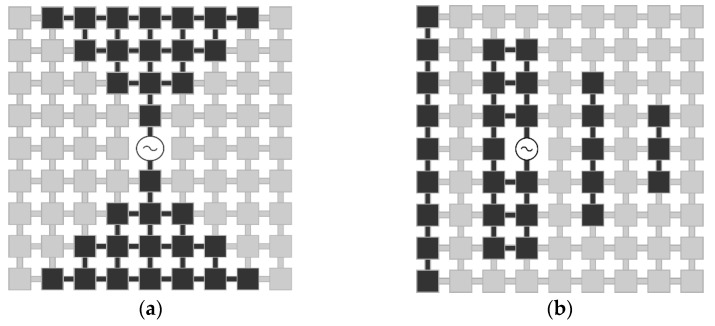
The shape-morphing capability of the pixel antenna is illustrated by representing different configurations ((**a**) bowtie pixel antenna (**b**) Yagi pixel antenna).

The most attractive compound reconfigurable antenna design is the pixel antenna [32,34]. The operation principle of pixel antennas is similar to that of an LCD screen, which can display arbitrary shapes [35]. Pixel antennas are usually composed of a two-dimensional grid, consisting of small metallic surfaces (patches) interconnected by sub-wavelength dimension switches, as displayed in Figure 2. By activating different configurations with the aid of the switches, the antenna surface is reshaped, hence the current is redistributed. The result is the alteration of the frequency and radiation characteristics of the antenna. This shape-morphing capability of pixel antennas provides a higher-level reconfigurability than other reconfigurable antenna architectures [28,32,36].

Generally, a microstrip antenna (MSA) in its simplest form consists of a radiating element of various shapes such as square, circular and triangular on one side of the substrate and ground plane on the other side [37,38]. Radiation of the MSA can occur from the fringing fields between the edges of the patch and the ground plane. Although a microstrip patch antenna has a compact structure and low profile, MSA suffers from drawbacks like narrow bandwidth, typically 1–5%, and low gain and is unsuitable for wide bandwidth applications [37,39]. One of the attractive methods to obtain broad bandwidth is to cut part of the ground plane from the back of the radiator patch and, in this case, it can be considered as planar monopole antenna. In this modification, loss of the directional radiation pattern feature of MSA can cause an omnidirectional radiation characteristic. The drawback of omnidirectional characteristics is that it leads to high interference and energy loss in unwanted directions at the expense of the main lobe energy [40]. Therefore, in this work we propose a planar monopole antenna due to its wide bandwidth and we focus on reconfigurable radiation characteristics to maximize the gain and power handling capability.

Although the principle of the pixel antenna was already demonstrated in [32,34] (pp. 3422–3427), (pp. 2219–2225), our proposed structure has several differences compared to the aforementioned references. In both references, the authors presented a pixel antenna working in the microwave region, while this work presents the behavior of the pixel antenna in the mid-IR region. The previous studies proposed RF switches controlled by external circuitry, while this work proposes bolometer switches incorporated into the arrangement, controlled directly by the incident wave.

## 3. Antenna Design

### 3.1. Reference Antenna

**Figure 3:**
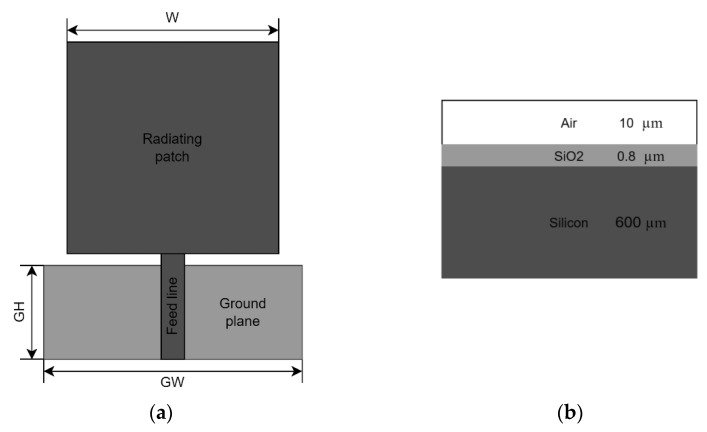
Reference planar antenna geometry (**a**), and substrate geometry (**b**) used for the simulations.

**Figure 4:**
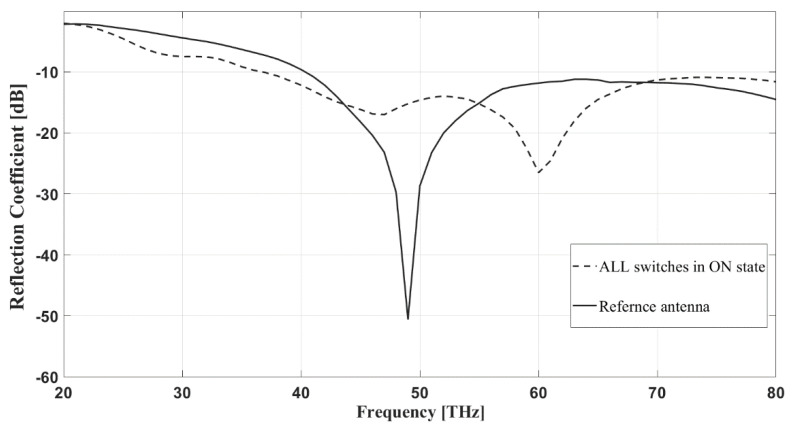
Reflection coefficients vs frequency in the case of the reference and pixel antennas. The simulations were done with the Sonnet Professional software.

**Figure 5:**
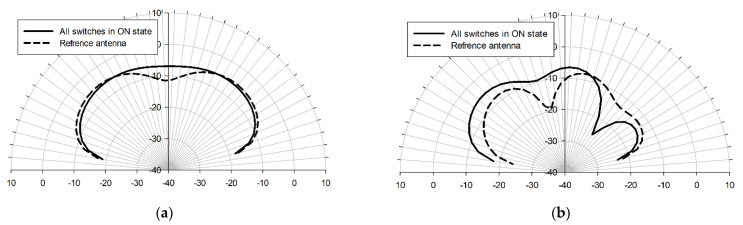
E-plane (xz-plane) (**a**) and H-plane (yz-plane) (**b**) radiation patterns (realized gain) of the reference and pixel antennas operating at 50 THz. The simulations were done with the Sonnet Professional software.

The first step of the procedure is the design of a reference planar monopole antenna (see Figure 3), optimized for the mid-IR band with the aid of the Sonnet Professional software. The square patch is placed on a ${\mathrm{SiO}}_{2}$ layer, supported by intrinsic silicon substrate with a thickness of 600 μm and ${є}_{r}=11.9$. The optimized thickness of the ${\mathrm{SiO}}_{2}$ layer is 0.8 μm, its relative permittivity is ${є}_{r}=3.9$. The side dimension of the square patch is W=4.5 μm. The antenna is fed from a 50 Ω microstrip line of 0.4 μm width, located on the ${\mathrm{SiO}}_{2}$ layer, as well. The feed line is backed by a rectangular ground plane of GW=4.9 μm, and GH=2 μm dimensions, placed at the interface of the Si and ${\mathrm{SiO}}_{2}$ layers. The matching between the feed line and patch is controlled by the ${\mathrm{SiO}}_{2}$ thickness and the size of the gap between the patch and ground, which is set to 0.2 μm. The frequency response of the reference antenna is displayed in Figure 4, which indicates that the antenna is well-matched for the mid-IR band. The radiation pattern of the antenna is displayed in Figure 5. It is uniform, since the antenna geometry is symmetrical with respect to the feed line, therefore the current distribution follows this symmetry, as well.

### 3.2. Pixelized Antenna

**Figure 6:**
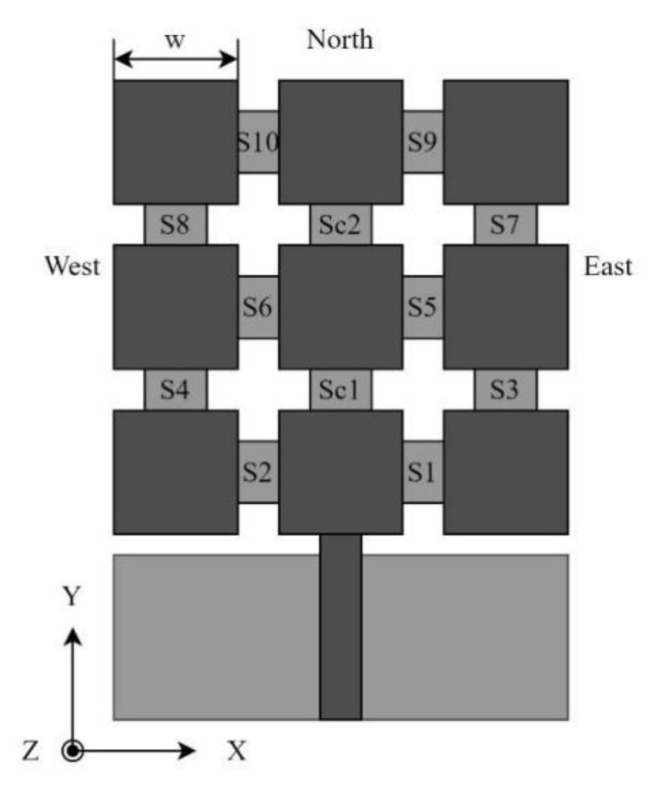
Pixel antenna geometry with the switches.

In the following, we present a pixel antenna design, which serves as the backbone of the proposed self-adapting antenna. The reference antenna is fragmented into 3×3 square pixels displayed in Figure 6. The pixel antenna structure consists of square patches, interconnected by small metallic parts serving as switches (a closed switch results in interconnected patches, an open one yields disconnected pixels). Each square pixel has w=1.5 μm side length, the gap between adjacent patches is 0.2 μm, and the dimensions of the metallic connectors are 0.4 μm ×0.2 μm.

The frequency response of the proposed pixel antenna is compared to that of the reference antenna when all of the pixels are interconnected (see Figure 4). The reflection coefficient of the two structures shows that both antennas work in the mid-IR band. Both the pixel and the reference antennas have a broadband frequency response with fractional bandwidth (FBW) of 62.5% and 45.6%, respectively.

The responses simulated with the Sonnet Professional software are similar, however, the bandwidth of the pixel antenna is slightly increased. The radiation patterns of the two structures at 50 THz are compared in Figure 5. Both arrangements have almost uniform radiation patterns in the E-plane direction, while the pixelized system earns around 2 dB gain on average and becomes more directive toward the pixels with respect to the ground (see Figure 5b).

### 3.3. Bolometer Switches

When IR radiation is incident on a bolometer, it heats up and its resistance changes. If its material has a positive temperature coefficient (PTC), its resistance increases with increasing temperature. On the other hand, a negative temperature coefficient (NTC) material operates in the opposite manner, like in the case of [37,38] (pp. 5818–5826) (pp. 827–828)), where a $V_{2}O_{5}$ bolometer is introduced. Furthermore, bolometers can be designed with characteristics dependent on the angle of incidence of the radiation [35,36]. In the absence of radiation, an NTC bolometer has high resistance, therefore it acts like an open switch if it is irradiated; due to the lower resistance it behaves like a closed switch. The behavior of PTC bolometers is the opposite.

Several materials with different temperature coefficients of resistance (TCR) can be used, such as nickel, niobium and vanadium oxide. In this work, we suppose the application of nickel bolometers described in [41] (pp. 5818–5826), with the inclusion of micro walls to control the directionality of the switches. Control of the incident wave on the switches can be achieved by using properly placed micro walls next to the bolometers in order to exploit the shadow effect produced by the walls, thereby making them directionally sensitive. The switches can be realized by presently available, conventional fabrication procedures.

In the following, we will show that if the interconnects between pixels are replaced by appropriate bolometers, the radiation pattern of the antenna can be made self-adjusting to the direction of the incident IR radiation. Figure 6 shows the schematic of the proposed pixel antenna-bolometer switch configuration, suitable for self-adjusting its radiation pattern to the direction of the incident mid-IR radiation. The design is optimized for the main directions (perpendicular, west, north, east). South was omitted due to difficulties related to the feed line and ground. The switches in the antenna arrangement are NTC bolometers, sensitive to radiation only from specific directions. The necessary directionalities of the switches are summarized in 

More than one switch arrangement can accomplish the task, and here we present two different solutions. If radiation is incident from the perpendicular direction, all of the bolometers switch to their ON state, and therefore all of the pixels are connected. The following simulation results will show (see later paragraphs) that this corresponds to a radiation pattern optimized towards the perpendicular direction. In the case of radiation coming from the northward direction, the IR radiation will activate the center switch Sc1, or Sc1 and Sc2 in the other possible configuration, and the characteristics of the antenna will lean toward the north (see next section for the simulation results). If the IR radiation hits from the west, S1 and S3, or, in the case of the other configuration, S1, S3, and S7 will be activated. This tilts the radiation pattern westward (see next section for the simulation results). On the other hand, when radiation is incident from the east, S2 and S4, or in the case of the other arrangement S2, S4, S8 will turn on, resulting in an eastward leaning antenna pattern (see next section for the simulation results). If radiation comes from multiple directions, most or all of the switches are activated making the antenna pattern more uniform.

## 4. Simulation Results

Both the operating frequency and radiation pattern of the antenna can be changed by adding or removing pixels from it using the proposed bolometer switches (see previous section). This section investigates various configurations of the pixel antenna with the aid of the Sonnet Professional antenna simulator software.

### 4.1. Center Pixels

**Figure 7:**
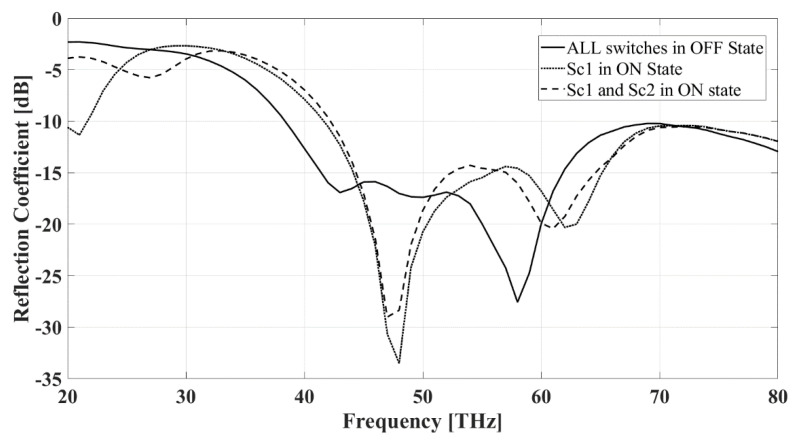
Reflection coefficient versus frequency when all of the switches are OFF, Sc1= ON, and Sc1 = Sc2 = ON. The simulations were done with the Sonnet Professional software.

**Figure 8:**
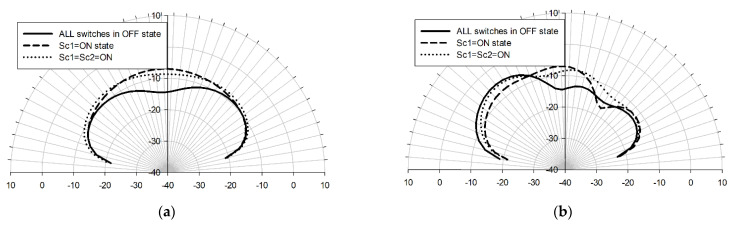
E-plane (xz-plane) (**a**) and H-plane (yz-plane) (**b**) radiation patterns (realized gain) when all of the switches are OFF (solid line), Sc1 = ON (dashed), and Sc1 = Sc2 = ON (dotted) at 50 THz. The simulations were done with the Sonnet Professional software.

If the incoming radiation is incident from the north, in the case of “arrangement 1”, Sc1 = ON, in the case of “arrangement 2”, Sc1 = Sc2 = ON (see Table 1). Figure 7 displays the frequency response of the antenna when all of the switches are in the OFF state (solid line), Sc1 = ON (dotted line), and Sc1 = Sc2 = ON state (dashed line), while all of the other switches are assumed to be in the OFF state. The frequency responses demonstrate a good matching with the mid-IR range in all of these three cases. The current distributions of the states are along the y-axis when the center switches Sc1 and Sc2 are activated. This change in current distribution results in a radiation pattern tilted northwards. Figure 8 displays the E- and H-plane patterns of the three different states. When Sc1 and Sc2 are activated, the antenna earns more than 5 dB gain in the perpendicular direction, as displayed in Figure 8a. On the other hand, the activation of these switches slightly steers the radiation pattern towards the north (y-axis), as presented in Figure 8b.

### 4.2. Symmetrical States

**Figure 9:**
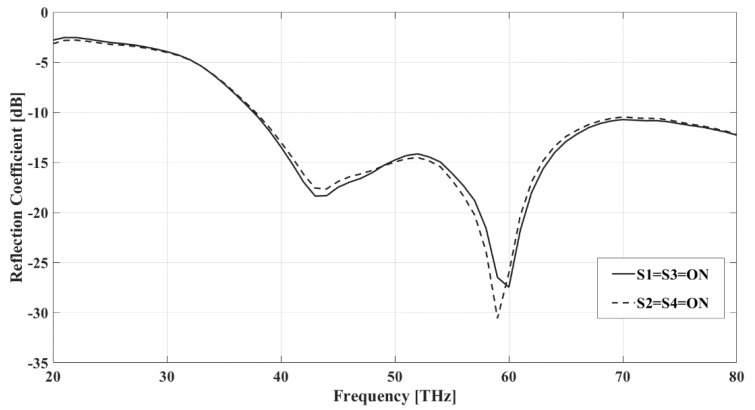
Reflection coefficient versus frequency when S2 = S4 = ON and S1 = S3 = ON. The simulations were done with the Sonnet Professional software.

**Figure 10:**
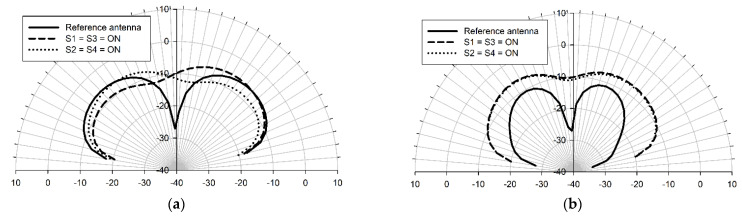
E-plane (xz-plane) (**a**) and H-plane (yz-plane) (**b**) radiation patterns (realized gain) of the reference antenna and pixel antenna, when S1 = S3 = ON and S2 = S4 = ON at 45 THz. The simulations were done with the Sonnet Professional software.

Configuring the antenna into symmetrical, mirror image-like patterns results in the mirroring of the current distribution through the pixels. Figure 9 displays the simulated return loss when S1 = S3 = ON (this occurs when radiation is incident from the west as per Table 1) and S2 = S4 = ON state (radiation is from the east per Table 1), while all of the other switches are in the OFF state. The frequency responses of the two conditions are identical since the physical lengths of the arrangements remain identical in both cases. Figure 10a shows that the E-plane patterns are the mirror images of each other. When S1 = S3 = ON, the radiation pattern leans towards the west, and when S2 = S4 = ON, the antenna pattern is eastward-directed. The mirrored current distributions explain this behavior. Figure 10b displays the identical H-plane patterns of the mirrored configurations and shows that the pixel antenna with these two states earns a considerable gain compared with the reference structure at 45 THz. Several other configurations can achieve the same effect when mirrored.

### 4.3. Additional Arrangements

**Figure 11:**
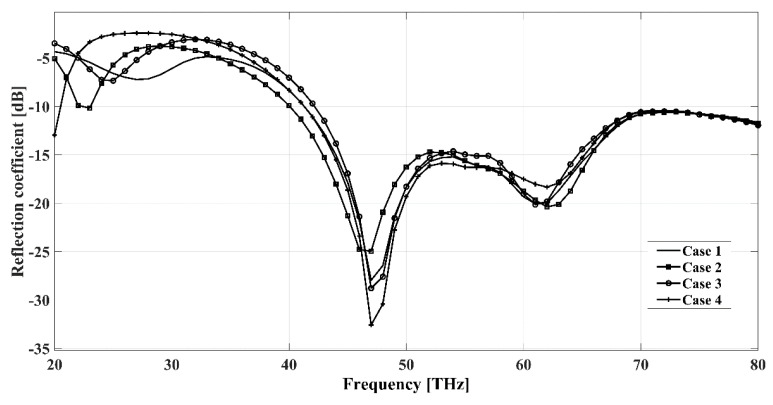
Reflection coefficients versus frequency of the pixel antenna when S2 = Sc1 = Sc2 = S9 = ON (case1), S1 = Sc1 = S6 = S8 = ON (case2), Sc1 = Sc2 = S9 = S10 = ON (case3) and Sc1 = S5 = S7 = S9 = S6 = S8 = S10 = ON (case4). The simulations were done wit. Reflection coefficients versus frequency of the pixel antenna when S2 = Sc1 = Sc2 = S9 = ON (case1), S1 = Sc1 = S6 = S8 = ON (case2), Sc1 = Sc2 = S9 = S10 = ON (case3) and Sc1 = S5 = S7 = S9 = S6 = S8 = S10 = ON (case4). The simulations were done with the Sonnet Professional software.

**Figure 12:**
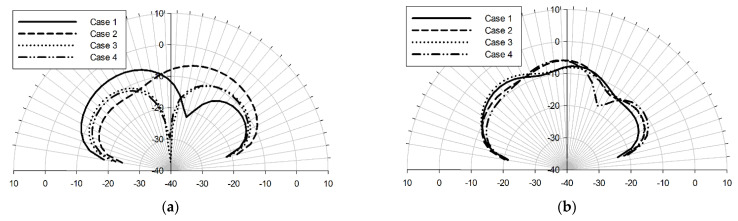
E-plane (xz-plane) (**a**) and H-plane (yz-plane) (**b**) characteristics of the pixel antenna when S2 = Sc1 = Sc2 = S9 = ON (case1), S1 = Sc1 = S6 = S8 = ON (case2), Sc1 = Sc2 = S9 = S10 = ON (case3) and Sc1 = S5 = S7 = S9 = S6 = S8 = S10 = ON (case4) at 50 THz.

In the following, we present the characteristics of four additional arrangements of the pixel antenna. Figure 11 shows the return loss of the antenna when S1 = Sc1 = S6 = S8 = ON, S2 = Sc1 = Sc2 = S9 = ON, Sc1 = S5 = S7 = S9 = S6 = S8 = S10 = ON and Sc1 = Sc2 = S9 = S10 = ON. We can observe that although the frequency responses slightly depend on the configuration, the antenna still operates in the mid-IR band in all of the cases. The E-plane and H-plane characteristics are displayed in Figure 12. The patterns show that besides the main directions described previously, the pixel antenna can be optimized for other in-between directions, such as perpendicular-west, and northwest.

## 5. Discussion

The simulation results suggest that the proposed adaptable pixel antenna arrangement possesses promising features suitable for mid-IR applications. The structure can automatically reconfigure its characteristics in order to optimize its properties for the incoming radiation, without the need for external circuitry and biasing. In other words, it is a self-adjusting system. The antenna has the benefit of increasing the collection area and minimizing the noise equivalent power (NEP), which is a critical issue in the case of IR detectors. Furthermore, it can capture the concentrated field coming from a specific direction in a much simpler and easier manner than the graphene based one previously introduced in the literature. Off-state pixels can work as parasitic elements serving as directors and/or reflectors, thereby increasing the collection area and the antenna gain. A wide variety of beneficial conditions can be achieved with the proposed structure, since it has $12^{2}=144$ possible states, however, it isn’t possible to explore all of them in a single paper. For example, the pixel concept could also provide dynamic matching between the antenna and rectifier, one of the most critical issues in an energy harvesting system. The proposed structure can be fabricated by involving thermal oxidation of the silicon wafer, optical and electron beam lithography, metal deposition and multiple developing and etching procedures [42].

## 6. Conclusions

In this paper, we presented a planar, monopole pixel antenna, combined with bolometer-based switches, self-adaptable to the direction of the incident radiation, suited for mid-IR frequencies. The proposed design possesses independent frequency and pattern reconfiguration capabilities in order to achieve maximum power handling in the case of various mid-IR applications. Moreover, the antenna arrangement earned a 2 dB gain. The pixelized structure achieved 25 THz bandwidth with 17% enhancement compared to the reference one. Since even in the case of the 3 × 3 pixel version there are 144 possible configurations, and the number of pixels can be further increased, there is a tremendous potential in such antennas regarding adaptable infrared applications. Such capabilities, the detailed fabrication process and investigation of the bolometer parameters will be explored in future works.

## Figures and Tables

**Table 1:** Directionalities of the bolometers in the pixel antenna structure.

**Arrangement 1**	
**Switch**	**Directionality**
S5, S6, S7, S8, S9, S10, Sc2	perpendicular
S2, S4	perpendicular and east
S1, S3	perpendicular and west
Sc1	perpendicular and north
**Arrangement 2**	
**Switch**	**Directionality**
S5, S6, S9, S10	perpendicular
S2, S4, S8	perpendicular and east
S1, S3, S7	perpendicular and west
Sc1, Sc2	perpendicular and north

## Data Availability

Not applicable.
